# Mast cell activation within the mesentery depends on vagal signaling following abdominal surgery

**DOI:** 10.1016/j.sopen.2026.03.005

**Published:** 2026-03-23

**Authors:** Anna Woestemeier, Timo Schwandt, Philipp Lingohr, Mariola Lysson, Sven Wehner, Jörg C. Kalff, Gun-Soo Hong

**Affiliations:** a Department for General, Visceral, Thoracic and Vascular Surgery, University Hospital of Bonn, Germany

**Keywords:** connective tissue mast cells, mast cell activation, postoperative ileus, vagus nerve, mesentery, POI, intestine

## Abstract

**Introduction:**

Abdominal surgery causes an inflammatory reaction in the intestines, resulting in a postoperative ileus. Mast cells can be divided into mucosal mast cells and connective tissue mast cells. In this study, we investigated mast cell activation within the gastrointestinal tract during abdominal surgery and explored the activation and role of connective tissue mast cells in postoperative ileus.

**Material and Methods:**

Postoperative ileus was induced in wild-type, Mcpt5-Cre xDTR^fl/fl^ and Mcpt5-Cre mice by intestinal manipulation. In vitro, vagal activation was further specified by stimulating mast cells with substance P, nicotine and bethanechol. Mast cell degranulation in the mesentery and stomach wall was visualized using avidin staining. Vagal requirement was assessed by performing unilateral cervical vagotomy prior intestinal manipulation. The role of macrophages was investigated by macrophage depletion before surgery. Intestinal inflammation was evaluated by analyzing leukocyte influx into the intestine, and motility was analyzed by a gastrointestinal transit after surgery.

**Results:**

Surgery induced mast cell degranulation within mesentery. Mast cell degranulation was independent of macrophage activity or mechanical stress, whereas vagotomy prevented mast cell activation in vivo. In vitro, mast cell degranulation was triggered by muscarinic acetylcholine receptors, but not by nicotinic receptors. However, Mcpt5-Cre DTR^fl/fl^ mice showed no alteration in leukocyte influx into the muscularis externa or gastrointestinal transit compared to controls.

**Conclusions:**

In conclusion, mast cell activation in the mesentery is induced via muscarinic acetylcholine receptors after surgery. However, depletion of these mast cells did not alter postoperative ileus in mice, suggesting that mast cells do not play a direct role in the pathogenesis of postoperative ileus.

## Introduction

Abdominal surgery leads to a transient phase of impaired bowel motility resulting in a postoperative ileus (POI) accompanied by vomiting, nausea, intolerance to food and delayed defecation [1]. This leads to a significantly increased morbidity for the patient and also causes increased health care costs [2], [3]. POI can be induced by intestinal manipulation (IM) during surgery, causing an immune-mediated condition, which activates resident macrophages within the muscularis externa [4]. Depletion of resident macrophages reduced the inflammatory response after IM and ameliorated the POI [5]. However, the role of mast cells during POI is controversial. Mast cell degranulation was associated with POI after surgery, mast cells deficient mice (Kit^W-sh/W-sh^) showed an improved gastrointestinal transit (GI-transit) after IM [6] and mast cell stabilizers prevented POI in a murine POI model [7], [8]. Also in a clinical setting, mast cell activation was related to GI recovery after abdominal surgery [9], [10]. However, the role of mast cells was challenged by Gomez-Pinilla et al. using Cpa3^Cre/+^ mice [11]. In a model of intestinal manipulation (IM), lack of connective tissue mast cells, mucosal mast cells, and a reduction of basophils in these mice [45] did not affect the postoperative infiltration of MPO^+^ cells in the tunica muscularis and the GI-transit compared to the control group. Yet, mast cell degranulation was observed after surgery and mast cell have been shown to be an important initiator of the innate immunity as well as modulating the adaptive immune response [12]. Many reports demonstrated a role of mast cells in host defense against pathogens [13], [14], [15] and also a key role during wound healing, tissue remodeling and transplant tolerance [16], [17], [18]. However, these studies primarily used Kit-mutant mice, which are now known to possess numerous phenotypic abnormalities beyond mast cell deficiency, including anemia, neutropenia, and gastrointestinal dysbiosis [50], [51]. Recent research using Kit-independent (or “Kit-native”) mast cell-deficient models has often failed to replicate these protective effects, suggesting that the susceptibility observed in older studies may have been due to Kit-related defects rather than the absence of mast cells [52]. Notably, mast cell populations are heterogeneous in the GI tract. Classically, mast cells are divided into connective tissue cells expressing Mcpt5, which are found within the mesentery, peritoneum and intestinal submucosa and mucosal mast cells are found within the intestinal mucosa [19], [20]. In this study, we first investigated the pathomechanism of mast cell degranulation and second, we used Mcpt5-Cre/DTR^fl/fl^ mice [21], in which only connective tissue mast cells were depleted without an impairment of the mucosal mast cells to investigate a subpopulation of mast cells in a murine POI model. In order to analyze the functional role of mast cell degranulation in mice in response to abdominal surgery we used different genetically modified mouse lines. Kit^W-sh/W-sh^ mice are completely devoid of mast cells in their gastrointestinal tract or other anatomical sites. These mice have been used before, but are rather unspecific in terms of the target mast cell population and also show reduced pacemaker cell levels in the gut. Mcpt5-Cre/DTR^fl/fl^ mice, however, have not been analyzed so far and show a distinct depletion of connective tissue mast cells. Since these cells reside in the mesentery and serosa they are the most likely candidates for initiating the hypothesized neuro-immune crosstalk. The use of Mcpt5-Cre/DTR^fl/fl^ mice, is essential for testing the hypothesis regarding vagal signaling. If vagal-cholinergic signaling targets connective tissue mast cells in the mesentery to trigger degranulation, a mucosal-deficient-only model or a broad-depletion model might mask this site-specific interaction.

We hypothesize that mesenteric mast cell activation following intestinal manipulation is regulated by vagal signaling through muscarinic acetylcholine receptors and contributes to postoperative ileus.

## Materials and methods

### Mice

Experiments were performed with 6–8 week old male wildtype C57BL/6 J mice (Janvier, France), Kit^W-sh/W-sh^ mice, Mcpt5-Cre/DTR^fl/fl^ mice with a mean body weight of 20-25 g. Mcpt5-Cre mice were crossed with inducible diphtheria toxin receptor (ROSA26iDTR) transgenic mic. The resulting Mcpt5-Cre/DTR^fl/f^ mice expressed the diphtheria toxin receptor (DTR) under the control of the Mcpt5 promoter. For Ablation of Mcpt5-expressing cells, we administered 25 ng diphtheria toxin per g bodyweight by intraperitoneal (i.p.) injection 24 h prior surgery. Only male mice were used as the immunological reaction can vary due to levels of progesterone and estrogens. All studies were approved by the committee for animal experiments of North-Rhine Westphalia (LANUV) and performed in accordance with federal law regarding the protection of animals. Animals were maintained on a 12-h light/dark cycle and provided with commercially available rodent chow and tap water ad libitum. The manuscript was written according to the Animal Research: Reporting In Vivo Experiments (ARRIVE) guidelines. To minimize bias and ensure the integrity of the data, mice were randomly assigned to experimental using a computer-generated random sequence. Personnel performing downstream assays were blinded to the surgical status of each animal.

### Operative procedures

Surgery was performed under aseptic conditions. Anesthesia was induced using isoflurane (Abbott, Wiesbaden, Germany) and oxygen as a gas carrier (1.5 l/min). Induction of anesthesia was achieved with 3% of isoflurane. The mouse's face was placed into a suitable mask during the surgery (approximately 20 to 30 min), under 1.5–3% of isoflurane. Mice received tramadol (Tramal, Grünenthal GmbH, Aachen, Germany) 30 mg/kg of body weight 1 h before and 2 h after surgery subcutaneously for analgesia. Afterwards, tramadol was dissolved within the drinking water (1 mg/ml) and mice were watered ad libiditum.

### Intestinal manipulation (IM)

Intestinal manipultation is a established model for POI [11], [43], [46]. Surgery was performed under aseptic conditions. Anesthesia was induced by 3% isoflurane (Abbott, Wiesbaden, Germany). The abdominal cavity was opened by a midline incision. Intestinal manipulation was performed twice for 5 min, the externalized small bowel was gently manipulated at a fixed distance of 1–2 cm from the distal duodenum to the cecum using sterile, moist cotton applicators while resting on a sterile moist gauze pad. The amount of pressure applied during gentle manipulation was approximately 90 mN to 100 mN [53]. Contact with the stomach or colon was avoided, as described previously [43].

The peritoneum was closed by a running suture using Vicryl 5–0 thread (Ethicon, Norderstedt, Germany), skin was closed with a running suture using Silk 5–0 (B.Braun, Melsungen, Germany). Control animals underwent laparotomy (lap) without IM procedure.

In the ex-vivo organ culture model, the mesenteric tissue was explanted and for 5 min the mesentery was gently manipulated from the distal duodenum to the cecum using moist cotton applicators.

### Vagotomy (VGX)

Animals were anesthetized as mentioned above. The skin was opened laterally to the thyroid and the right cervical vagal nerve was carefully prepared from the carotid artery. A 2–3 mm piece of the vagal nerve was dissected by fine micro scissors. The skin was closed by a running suture. Control animals underwent exploration of the vagal nerve without dissection (–VGX).

### Post-surgery care

After surgery, mice were treated with analgesia as described before. They were placed in cages that were placed under an infrared lamp during 2–3 h post-surgery. Afterwards, they were housed again under specific pathogen-free conditions including a 12/12 h light/dark cycle, 21 °C and 30% relative humidity in the animal housing facility of the University of Bonn (Germany).

### Animal euthanasia

After indicated time points, animals were sacrificed for further analysis. Euthanasia was performed by cervical dislocation.

### Detection of myeloperoxidase^+^ (MPO^+^) leukocytes

Jejunal muscularis whole mounts underwent myeloperoxidase staining as described previously [22]. In short, muscularis whole mounts were fixed with 100% ethanol for 10 min at room temperature (RT). After washing with krebs-henseleit buffer, jejunal muscularis were mechanically separated from mucosa and specimen were incubated in a sodium acetate buffer (pH 5.0) containing 10% 3-amino-9-ethyl carbazole (Polysciences, Warrington, USA) and 0,03% H2O2. Specimens were cover slipped in aqueous mounting medium and inspected by light microscopy. MPO^+^ leukocytes were microscopically counted in 5 randomly chosen areas at a magnification of 200×.

### Macrophage depletion

Mice were given 1 ml Cl_2_MDP liposomes (50 mg chlodronate/kg) per 100 g body weight at a size of 400 nm on days −2 (before the operative procedure). To ensure effective depletion, CD11b^+^F4/80^+^ macrophages of the peritoneal cavity were analyzed via flow cytometry (Supplemental Fig. 1a).

### Preperation of liposomes

Cl_2_MDP liposomes were prepared according to the protocol of van Rooijen and Sanders [23]. In brief, 86 mg L-α-phosphatidylcholin and 8 mg cholesterol were dissolved in chloroform, vacuum evaporated and dissolved in a clodronate solution (2.5 g clodronate/10 ml aqua dest.). After water bath sonication non-encapsulated clodronate was removed from the suspension by centrifugation (10,000 for 15 min). The Cl_2_MDP liposomes that formed a band were washed twice with PBS and resuspended in 4 ml PBS for injection. The Cl_2_MDP-liposome suspension contained about 5 mg clodronate/ml.

For further experiments, liposomes were extruded with a Mini-Extruder through a polycarbonate membrane of pore size 400 or 100 nm (Avanti Polar Lipids, Alabaster, Alabama, USA). The liposome radius was measured by dynamic light scattering using ALV-NIBS/HPPS (high-sensitivity version) and the ALV-NIBS/HPPS V.0.3.0 software (ALV-GmbH, Langen, Germany).

### Staining of mast cells by avidin-biotin-peroxidase complexes (ABC)

#### Mesentery

The mesentery was dissected and mechanically fixed on a Sylgard-filled dish in DMEM +10% fetal calf serum (FCS) on ice. To analyze the induction of mast cell activation, mesentery tissue was incubated either in substance P, nicotine or bethanechol in different concentrations for 1 h at 37 °C. After washing the tissue with phosphate-buffered saline (PBS) three times, the tissue was blocked (PBS + 3%BSA + NaN3) for 30 min at room temperature. Next, it was incubated with Avidin (Avidin, Texas Red™ conjugate, Thermo Fisher Scientific, Waltham, USA) 1:1000, washed three times with PBS and fixated with formaldehyde 4% (Carl Roth, Karlsruhe, Germany). Afterwards, the tissue was washed with PBS three times and in Aqua dest. for 1 min. Mast cells were counted and graded (grade 1: no degranulation; grade 2: few granules extracellular; grade 3: approximately 50% granules extracellular, grade 4: > 75% granules extracellular) in at least 5 mesenteric arcades per mouse at a magnification of x200 (Supplemental Fig. 1b,c).

#### Intestine

After dissection of the intestine, tissue was embedded in Tissue-Tek (Sakura Finetek Inc., Torrance, CA), and cryostat-cut. Sections were washed twice with PBS and blocked (PBS + 3%BSA + NaN3) for 30 min. at room temperature. Afterwards, it was incubated with Avidin (Avidin, Texas Red™ conjugate, Thermo Fisher Scientific, Waltham, USA) 1:1000, washed three times with PBS and fixated with formaldehyde 4% (Carl Roth, Karlsruhe, Germany). Next, sections were washed with PBS three times and washed in Aqua dest. for 1 min.

### Drugs and solutions

KHB was used with the following constituents (concentrations expressed as mmol/L): Na+, 137.4; K+, 5.9; Ca++, 2.5; Mg++, 1.2; Cl--, 134; HCO3-, 15.5; H2PO4-, 1.2; and glucose, 11.5. All chemicals used for this study (if not separately mentioned) were purchased from Sigma Aldrich (Taufkirchen, Germany).

### Measurement of the gastrointestinal transit (GI-transit)

GI-transit was measured 24 h after IM and lap. A FITC-labelled dextran (70 kDa) was gavaged 90 min before organ harvest. The GI tract was divided into 15 segments (1× stomach, 10× small bowel, 1× ceceum, 3× colon) and luminal contents were washed out to determine the fluorescence at 494/521 em/ex. The geometric center was calculated by the following formula: GC = Σ (% of total fluorescent signal per segment*segment number) / 100.

### Tryptase release

To assess the activation of mast cells in response to intestinal handling, the expression and release of tryptase, a prestored mast cell-specific protease (Hogan, A. Det al.), was analyzed. The peritoneal cavity was flushed with 1 ml PBS after the indicated time points after IM or lap and the solution was aspirated with a syringe. The total tryptase (α-protryptase and β-tryptase) concentration per pg/1 ml peritoneal lavage fluid was measured in peripheral blood and lavage fluid samples using ELISA Kit; Biovenic, NY, USA.

### Statistics

Statistical analysis was performed with Prism V5.04 (GraphPad, San Diego, CA) using one-way analysis of variance with Bonferroni post-hoc test, two-way analysis of variance with Bonferroni post-hoc test or Student *t-*test as indicated and displayed as means+SEM. Data were considered statistically significant at *p*-values <0.05 (*), <0.01 (**) and < 0.001 (***).

## Results

### IM induces mast cell degranulation in the mesentery, but not within the bowel wall

**Fig. 1:**
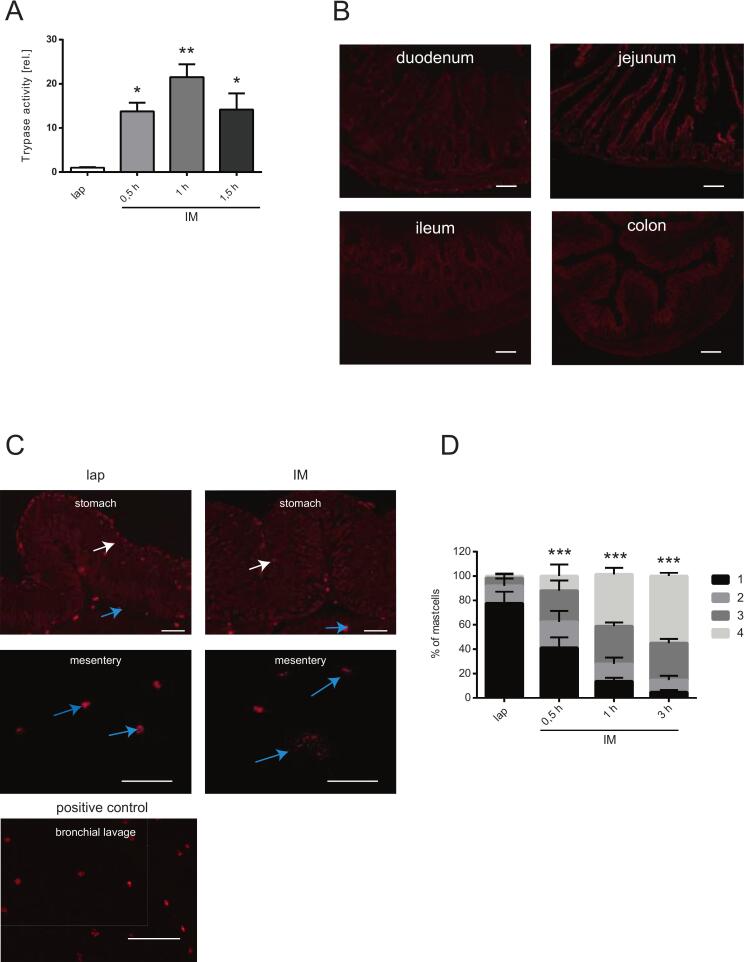
Mast cell degranulation was induced by IM in the mesentery, but not within the gastrointestinal tract (A) Mice underwent intestinal manipulation (IM) or laparotomy (lap), tryptase activity was measured after different time points within peritoneal lavage. (B) Representative micrographs of mast cell stainings by avidin-biotinylated peroxidase complexes within the duodenum, jejunum, ileum and colon within naïve mice. Micrographs were taken at 200× magnification and are representative for five independent experiments. Bars = 100 μm. (C) Representative micrographs of mast cell staining by avidin-biotinylated peroxidase complexes within the stomach and mesentery after laparotomy (lap) or IM (white arrow: mucosal mast cells; blue arrows: connective tissue mast cells). Micrographs were taken at 200× magnification and are representative for five independent experiments. Bars = 100 μm. (D) IM-induced mast cell degranulation (grade 1: no degranulation; grade 2: few granules extracellular; grade 3: approximately 50% granules extracellular, grade 4: > 75% granules extracellular) was measured after different time points within the mesentery. Statistical analysis was performed by a two-way ANOVA followed by Bonferroni's post test and displayed as means+SD of 5 mice per group. * = *p* < 0.05, **p < 0.01 and *** = *p* < 0.001 vs. lap. (For interpretation of the references to colour in this figure legend, the reader is referred to the web version of this article.)

In order to analyze, whether mast cell degranulate upon abdominal surgery, we explored a peritoneal lavage fluid for mast cell tryptase activity at different time points after IM. Tryptase activity was significantly increased at 0.5 h (*p* < 0.05), 1 h (*p* < 0.01) and 1.5 h (p < 0.05) after IM compared to lap (Fig. 1A). Next, the location of degranulated mast cells was identified by avidin staining within cross sections of different regions of the GI tract and within whole mount specimens of the mesentery. The avidin staining revealed that mast cells within the duodenum, jejunum, ileum or colon are rare compared to mucosal and connective tissue mast cells were detected within the stomach (Fig. 1B/C). Connective tissue mast cells were also found within the mesentery. Interestingly, IM did not increase mast cell degranulation in the gastric tissue (Fig. 1C). while mesenteric mast cells clearly degranulate immediately after IM (0.5 h (*p* < 0.001); 1 h (p < 0.001) and 3 h (p < 0.001)) compared to lap (Fig. 1C/D).

### Mast cell degranulation depends on vagal signaling, but is independent of peritoneal macrophages or mechanical stress

**Fig. 2:**
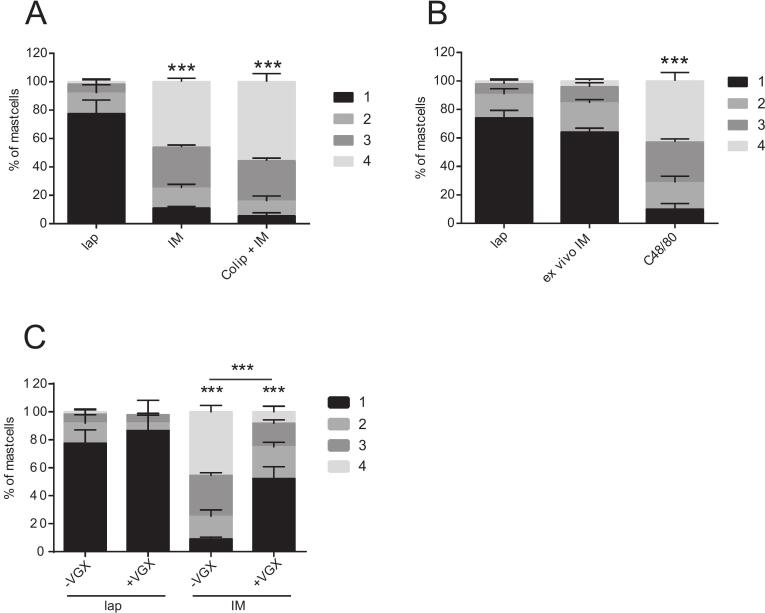
Mast cell degranulation depends on vagal signaling, but is independent of peritoneal macrophages or mechanical stress Mast cell degranulation (grade 1: no degranulation; grade 2: few granules extracellular; grade 3: approximately 50% granules extracellular, grade 4: > 75% granules extracellular) was measured after (A) laparotomy (lap), intestinal manipulation (IM) and macrophage depletion prior IM (Clolip + IM). (B) Mast cell degranulation was measured after lap, ex vivo manipulation of the intestine (ex vivo IM), C48/80 serve as a positive control. (C) Vagotomy (+VGX) or sham vagotomy (-VGX) was performed seven days before lap or IM. Statistical analysis was performed by a two-way ANOVA followed by Bonferroni's post test and displayed as means+SD of 10 mice per group. * = *p* < 0.05, **p < 0.01 and *** = *p* < 0.001 vs. lap or indicated otherwise.

As mast cell degranulation can be induced by a wide variety of stimuli including inflammatory mediators, allergens, pathogens, neurotransmitters and physical stress [24], [25], [26], [27]. Therefore, we tried to identify the mechanism which led to mesenteric mast cell degranulation after IM. We first explored the role of the resident macrophages as these cells can release factors that trigger mast cell degranulation as a consequence. Peritoneal macrophages were depleted with clodronate liposomes prior to IM. However, macrophage-depleted mice did not show any alteration in mast cell degranulation compared to the control group with no prior macrophage depletion (Fig. 2A).

Next, the mechanical stress that is applied during abdominal surgery was tested as an activator for mast cell degranulation. Therefore, we manipulated the mesenteric tissue in an ex vivo organ cultures model. However, the mesenteric harvesting procedure alone, as well as the ex vivo mechanical manipulation to the mesentery did not induce mast degranulation compared to control tissue that was only extracted and incubated in plain medium (Fig. 2B).

Neurotransmitters are further activators of mast cells and as vagal nerve terminals were described prior within the immediate environment of mast cells in the mesentery [28], a vagal influence during mast cell degranulation within the mesentery was explored by VGX performed seven days before further treatment.

IM-induced mast cell degranulation was significantly elevated in –VGX mice (*p* < 0.001) and VGX mice (p < 0.001) compared to the associated laparotomy control groups. However, IM-induced mast cell degranulation was ameliorated after VGX compared to -VGX animals (p < 0.001) (Fig. 2C). This indicated that vagal activation induces mesenteric mast cell degranulation during IM, but not direct mechanical stress or macrophage derived factors. These findings may point to an auxiliary stimulus for mast cell degranulation or, alternatively, represent the effects of the unilateral vagotomy.

### Mast cell degranulation in the mesentery is activated by muscarinic acetylcholine receptor

**Fig. 3:**
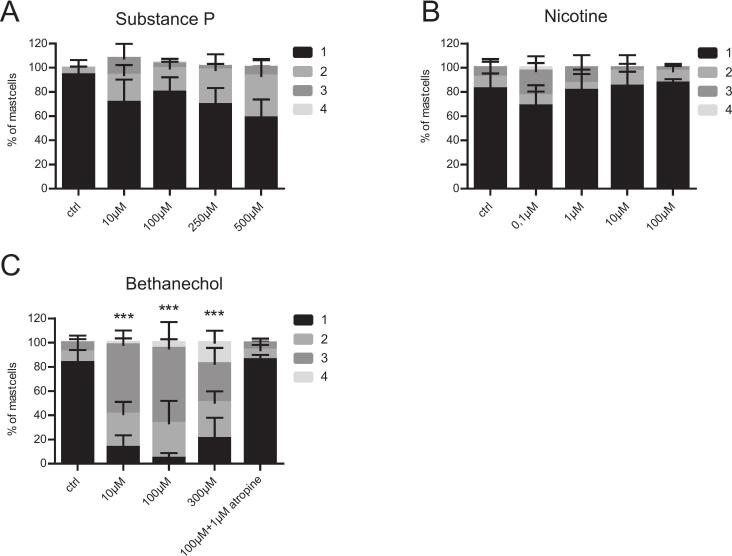
Mast cell degranulation in the mesentery can be induced via muscarinic receptor Mesentery tissue of naïve mice was removed to measure mast cell degranulation (grade 1: no degranulation; grade 2: few granules extracellular; grade 3: approximately 50% granules extracellular, grade 4: > 75% granules extracellular) after incubation in DMEM + P/S + 10% FCS (ctrl) compared to (A) substance P (B) nicotine or (C) bethanechol in different concentration. The experiment was replicated five times. Statistical analysis was performed by a two-way ANOVA followed by Bonferroni's post test and displayed as means+SD. * = p < 0.05, **p < 0.01 and *** = p < 0.001 vs. ctrl.

As a VGX prevented mast cell activation, we next investigated if vagal neurotransmitters trigger mesenteric mast cell degranulation ex vivo. Therefore, organ cultures of explanted mesenteric tissue were stimulated with increasing concentrations of the following vagal neurotransmitters: substance P, and the cholinergic agonists nicotine and bethanechol, which activate different subsets of cholinergic receptors (neurokinin receptor, nicotinic acetylcholine receptor, muscarinic acetylcholine receptor). These mediators directly stimulate mast cells, but their receptors are also widely distributed across various other cell types within the intestine and mesentery [54]. No significant mast cell degranulation was detected after incubation with substance P or nicotine (Fig. 3 A/B) while incubation in bethanechol, predominantly activating muscarinic cholinergic receptors, led to a significant increase of mast cell degranulation compared to the control tissue (10 μM: *p* < 0.001; 100 μM: p < 0.001), 300 μM: p < 0.001). Consequently, this effect could be antagonized by the addition of the muscarinic antagonist atropine (Fig. 3C). Our results demonstrate that mesenteric mast cell degranulation is induced in vivo by a vagus-nerve dependent mechanism which most likely directly activated by muscarinic receptors on mast cells.

Inflammatory response within ME and GI-transit after IM are improved in Kit^W-sh/W-sh^ mice, but not in Mcpt5-Cre/DTR^fl/fl^ mice compared to Bl6 mice.

**Fig. 4:**
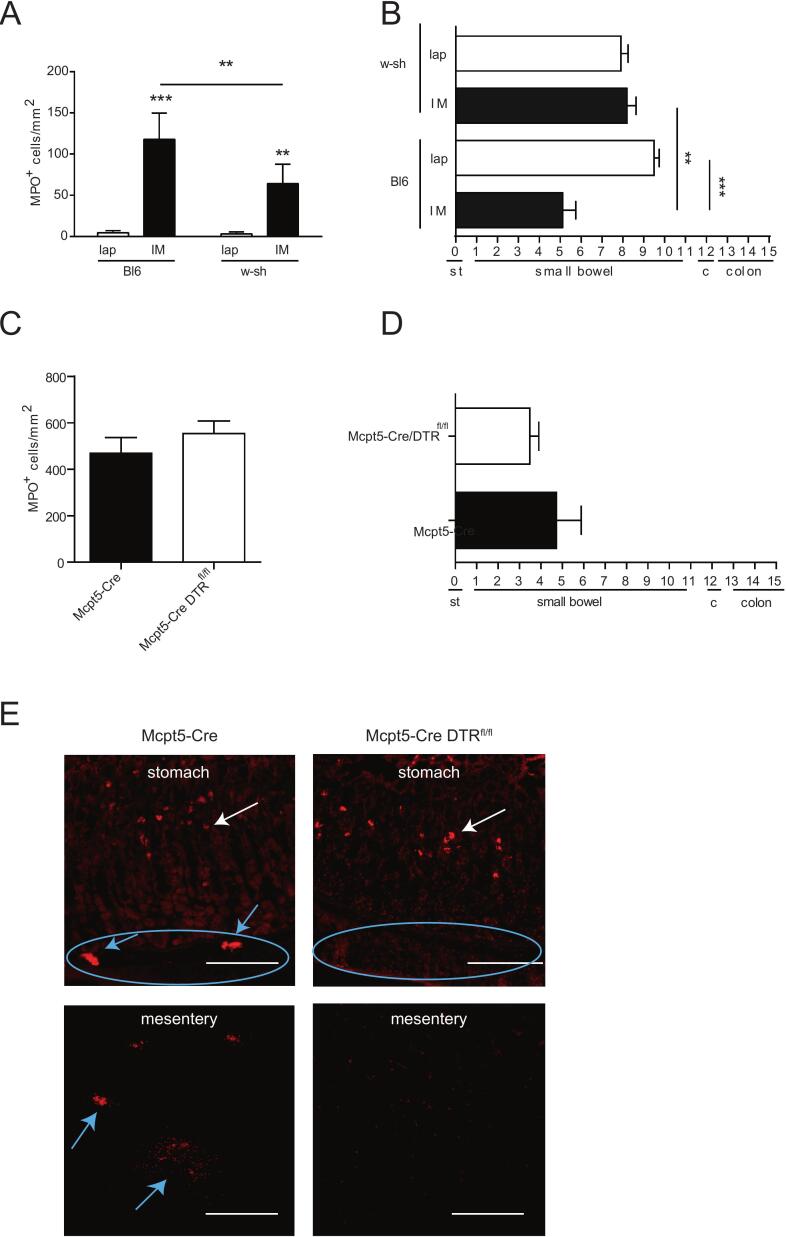

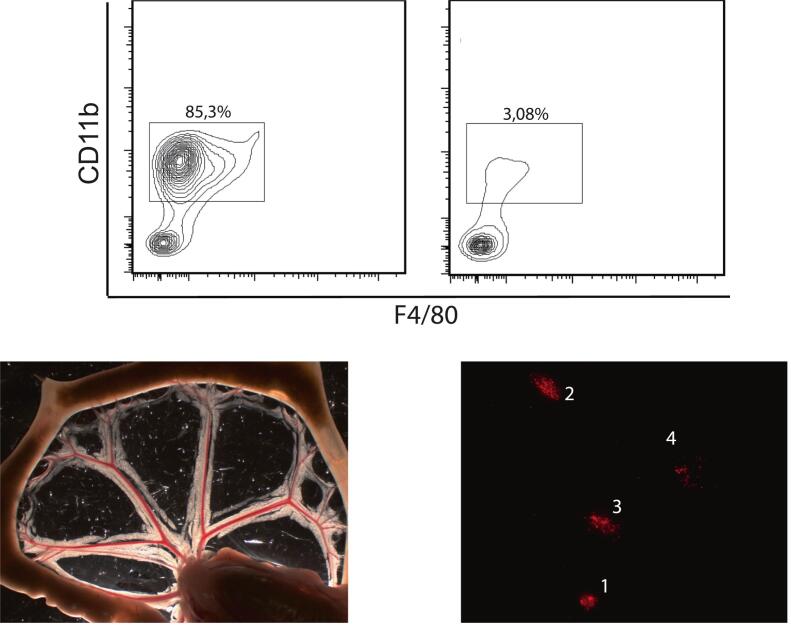
Influx of MPO^+^ cells and GI-transit after IM are independent of connective tissue mast cells Kit^W-sh/W-sh^, Mcpt5-Cre and Mcpt5-Cre DTR^fl/fl^ mice underwent laparotomy (lap) or intestinal manipulation (IM). MPO^+^ cells within the small bowel muscularis externa (A + C), and GI-transit (B + D) were determined 24 h after surgery. (E) Representative micrographs of mast cell stainings by avidin-biotinylated peroxidase complexes of Mcpt5-Cre and Mcpt5-Cre DTR^fl/fl^ mice after IM (white arrow: mucosal mast cells; blue arrows: connective tissue mast cells). Micrographs were taken at 200× magnification and are representative for five independent experiments. Bars = 100 μm. Statistical analysis was performed by a two-way ANOVA followed by Bonferroni's post test and displayed as means+SD of 10mice per group. * = *p* < 0.05, **p < 0.01 and *** = *p* < 0.001 vs. lap or indicated otherwise. (For interpretation of the references to colour in this figure legend, the reader is referred to the web version of this article.)

As shown before, leukocyte extravasation was significantly increased after IM compared to a laparotomy in Kit^W-sh/W-sh^ mice (*p* < 0.01) and in Bl6 mice (*p* < 0.001) (Fig. 4A). However, Kit^W-sh/W-sh^ mice showed a reduced leukocyte infiltration after IM was compared to Bl6 mice (p < 0.01). Functionally, IM-induced GI dysmotility was significantly pronounced in Bl6 mice after IM (p < 0.01) while Kit^W-sh/W-sh^ mice transit times were not reduced (Fig. 4B).

In Mcpt5-Cre/DTR^fl/fl^ mice, we first confirmed depletion of connective mast cells within the gut wall and mesentery, while mucosal mast cell levels are unaffected (Fig. 4 A,C + E). Although we have shown that mesenteric mast cells degranulate after IM, Mcpt5-Cre/DTR^fl/fl^ did neither show a difference in IM-induced infiltration of MPO^+^ cells into ME nor an alteration in GI-transit times compared to Mcp5-Cre control mice (Fig. 4A+B).

Together these data show that connective tissue mast cells in the mesentery immediately degranulate upon an abdominal surgical trauma via a muscarinic-vagus nerve triggered mechanism. However, connective tissue macrophages do not contribute to the postoperative immune response and functional motility disturbances in the GI tract.

## Discussion

Current understanding indicates that mast cell activation in the mesentery is a significant contributor to the inflammatory response following abdominal surgery [38]. The vagal nervous system, with its capacity to both sense and modulate inflammation, plays a crucial role in the postoperative period. Evidence from related models, such as functional dyspepsia and stroke, suggests a potential interplay between vagal signaling and mast cells [39], [40], [41]. However, there is a notable gap in research directly demonstrating the dependence of mesenteric mast cell activation on vagal signaling specifically after abdominal surgery. The role of mast cells during postoperative ileus has been discussed controversially [7], [8], [11]. The activation of peritoneal mast cells and mastcell activation in the mesentery has been described by Gomez-Penilla before [11]. As the manipulation of the intestine leads not only to a local inflammation, but also causes neural reflexes [47] with an activation of vagal nerves, mast cell activation in the mesentery is possible [46]. As shown before in mice [7] and humans [9], [10] mast cell activation was significantly elevated in the peritoneal lavage immediately after IM, indicating a mast cell activation by surgery. Even though mast cells were observed within the intestine before [7], we showed mucosal mast cells and connective tissue mast cells within the stomach wall and connective tissue mast cells in the mesentery, but did not detect a significant amount of mucosal or connective tissue mast cells within the intestine. Most mast cell stainings were performed using toloudine, which is also positive for mucin, amyloids, *Helicobacter pylori* and granules of endocrine cells. Mast cells have diverse functions due to their origin, differentiation and location [29]. In the gastrointestinal tract, there are two subtypes: the connective tissue mast cells and mucosal mast cells. The former used W/kit mice [1], [7], that not only lack mast cells, but the W/kit gene is required for interstitial cells of Cajal and for intestinal pacemaker activity. Gomez-Pinilla et al. used a genetically modified mouse strain with targeted insertion of Cre-recombinase into the caboxypepitdase A3, Cpa3^Cre/+^mice, where mast cells are ablated more specifically, but still both subtypes were absent [11]. As we observed mast cell degranulation selectively within connective tissue mast cells and not mucosal mast cells, we used for the first time Mcpt5-Cre/DTR^fl/fl^, where mast cells are ablated by a strategy utilizing the promoter for Mcpt5, a chymase that is highly restricted to mast cells of the connective tissue mast cell subtype [21]. Even though mesenteric mast cells have been shown to induce an inflammatory response in a LPS model previously [30], activation of mast cell degranulation within the mesentery has not been further explored until now. Here, we showed that unlike the resident macrophages within the muscularis externa of the intestine [31], mesenteric mast cells could not be activated by mechanical stress alone, indicating a different pathway to activate mast cells. Mucosal mast cells have rarely been shown within the intestinal wall. The immunohistochemical identification of mast cells in the intestine relied heavily on c-kit (CD117) expression; however, this marker is also expressed by interstitial cells of cajal (ICC), hematopoietic stem cells, and other lineages [11]. To ensure histological accuracy and avoid the overcounting of c − Kit+ cells as mast cells, contemporary studies—particularly those focusing on human mucosal mast cells—now utilize Anoctamin-1 (Ano-1) as a specific counterstain to definitively distinguish the ICC population from the resident mast cell pool [11]. Furthermore, mast cell activation was not measured within the tissue, but in the peritoneal cavity or even serum [48], [49]. Additionally, a clinical study showed no difference in mast cell activation depending on the surgical approach (open vs. minimally invasive colectomy) indicating that the severity of trauma is not crucial for mast cell activation [9]. Next, macrophages were depleted prior to IM to explore the role of macrophages during mast cell activation as macrophages are a well-known activator of mast cell degranulation [32]. We did not detect any mast cell activation alteration after macrophage depletion, leading us to conclude macrophage-independent mast cell activation. Even though macrophages can also have regulatory functions in mast cell activation, this could still be relevant in the mesentery during surgery [33].

Mast cell activation within the gastrointestinal tract has been shown to also depend on vagal signaling [34]. Additionally, the close anatomical proximity of vagal nerve endings and mast cells in the mesentery [28] could cause a paracrine activation. In this study, we observed vagus nerve-dependent mast cell degranulation during POI. However, vagotomy (VGX) did not completely prevent mast cell degranulation after IM compared to laparotomy. This could be due to the spared left vagus nerve, as a bilateral cervical VGX would be lethal.

Efferent vagal nerve fibers can modulate inflammatory processes within the mesentery through the release of the neurotransmitter acetylcholine [42]. Interestingly, mucosal mast cells have been shown to be inhibited by the vagus nerve via the nicotinic acetylcholine receptor [35], confirming our findings that mast cell degranulation could not be induced by nicotine but is mediated by the muscarinic acetylcholine receptor. There is even evidence that afferent fibers of the vagal nerve are activated by mast cell degranulation, which could lead to a feedback effect [36]. This could indicate a regulatory function of the vagus nerve in the activation of mast cells [19]. However, depletion of connective tissue mast cells in Mcpt5-Cre DTR^fl/fl^ mice did not prevent POI after IM compared to controls, whereas POI was prevented in Kit^W-sh/W-sh^ mice. This confirms previous findings that Kit^W-sh/W-sh^ are not an appropriate mouse model to investigate the role of mast cells in gastrointestinal diseases [11], as the W/kit gene is required for interstitial cells of Cajal and for intestinal pacemaker activity [37]. While Gomez-Pinilla et al. utilized a model that eliminates all mast cell types, we employed a second model—deficient specifically in connective tissue mast cells—to further validate these findings.

A principal limitation of this study is the use of a unilateral cervical vagotomy, which does not completely abolish vagal efferent and afferent signaling and therefore cannot fully exclude residual autonomic modulation of mesenteric mast cell activity. In addition, the genetic models employed to manipulate mast cell populations have inherent constraints: Kit^W-sh/W-sh^ mice lack mast cells but also exhibit deficiencies in interstitial cells of Cajal and impaired intestinal pacemaker activity, while Mcpt5-Cre/DTR^fl/fl^ mice selectively deplete connective tissue mast cells but leave mucosal mast cells and other immune cell populations intact. Consequently, these off-target and subset-specific effects may confound interpretation of mast cell–dependent mechanisms, and, together with known interspecies differences in mast cell distribution and phenotype between mice and humans, limit the direct translational extrapolation of our findings to the clinical setting. Furthermore, murine mast cell distribution and receptor expression profiles differ from those in humans, restricting direct translational applicability. Thus, while mechanistic insights into vagal regulation of mast cell activation are advanced, extrapolation to the human postoperative condition should be undertaken with caution.

In conclusion, abdominal surgery induces muscarinic acetylcholine receptor–dependent activation of mesenteric connective tissue mast cells, but depletion of this subset does not modify postoperative ileus in mice. These findings, in line with previous work [11], suggest that mesenteric connective tissue mast cells are unlikely to be the primary drivers of POI.

## CRediT authorship contribution statement

**Anna Woestemeier:** Writing – review & editing, Writing – original draft, Methodology, Investigation, Formal analysis, Data curation, Conceptualization. **Timo Schwandt:** Writing – review & editing, Validation, Methodology, Investigation, Formal analysis. **Philipp Lingohr:** Writing – review & editing, Data curation. **Mariola Lysson:** Writing – review & editing, Formal analysis. **Sven Wehner:** Writing – review & editing, Resources, Conceptualization. **Jörg C. Kalff:** Writing – review & editing, Resources, Funding acquisition. **Gun-Soo Hong:** Writing – review & editing, Writing – original draft, Project administration, Methodology, Investigation, Conceptualization.

## Ethics approval

All studies were approved by the committee for animal experiments of North-Rhine Westphalia (LANUV) and performed in accordance with federal law regarding the protection of animals. The manuscript was written according to the Animal Research: Reporting In Vivo Experiments (ARRIVE) guidelines.

## Disclosure

MCPT5-Cre mice were kindly provided by Axel Roers from the Institute for Immunology of the university of Dresden.

## Funding

This study was supported by a grant from 10.13039/501100001659DFG (KA 1270/5-1, grant to J. C. Kalff).

## Declaration of competing interest

SW and JCK received royalties from Wolters Kluwer.

## Appendix A Supplementary data

Suppl. Fig. 1 (A) Representative flow cytometry analysis to ensure effective macrophage depletion. CD11b^+^F4/80^+^ macrophages of the peritoneal cavity were analyzed via flow cytometry. (B) Representative image of mesentery tissue taken at 4× magnification. (C) Representative micrographs of mast cell staining by avidin-biotin-peroxidase complexes (ABC) and assessment of degranulation (Grade 1: no degranulation; grade 2: few granules extracellular; grade 3: approximately 50% granules extracellular, grade 4: > 75% granules extracellular). Micrographs were taken at 200× magnification. Image 1
