# Supplementary figures and images for: Mast cell activation within the mesentery depends on vagal signaling following abdominal surgery
Source: Surg Open Sci. 2026 Mar 23;31:44–53. doi: 10.1016/j.sopen.2026.03.005 (PMC13054006; doi:10.1016/j.sopen.2026.03.005)

A

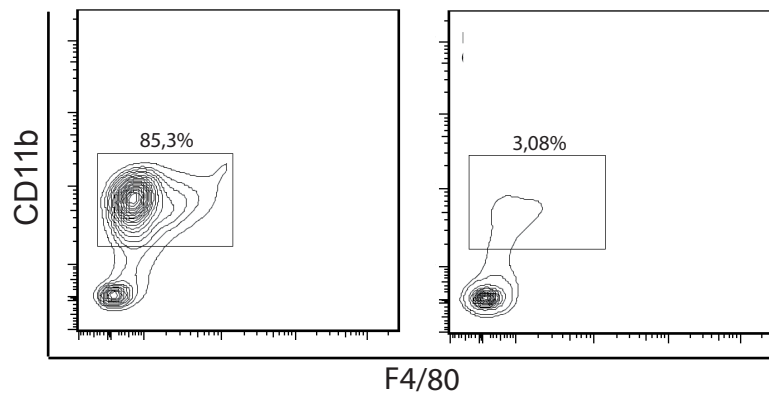

B

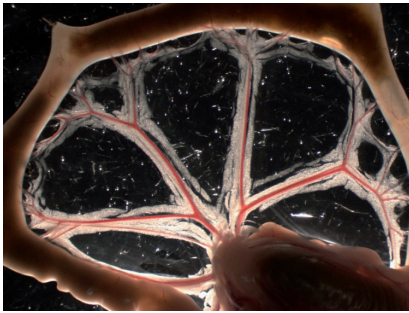

C

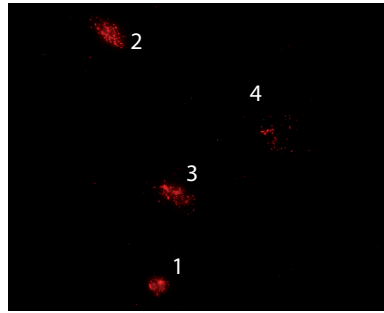

Supplement: Suppl. Fig. 1 — (A) Representative flow cytometry analysis to ensure effective macrophage depletion. CD11b+F4/80+ macrophages of the peritoneal cavity were analyzed via flow cytometry. (B) Representative image of mesentery tissue taken at 4× magnification. (C) Representative micrographs of mast cell staining by avidin-biotin-peroxidase complexes (ABC) and assessment of degranulation (Grade 1: no degranulation; grade 2: few granules extracellular; grade 3: approximately 50% granules extracellular, grade 4: > 75% granules extracellular). Micrographs were taken at 200× magnification. [file mmc1.pdf]
